# Supplementary material for: Extracellular Vesicle-Mediated Delivery of AntimiR-Conjugated Bio-Gold Nanoparticles for In Vivo Tumor Targeting
Source: Pharmaceutics. 2025 Aug 5;17(8):1015. doi: 10.3390/pharmaceutics17081015 (PMC12389129; doi:10.3390/pharmaceutics17081015)
Supplement: Supplementary file 1 [file pharmaceutics-17-01015-s001.zip › pharmaceutics-3762776-supplementary.pdf]

Supplementary data

Bio produced nanoparticles load into extracellular vesicles and support horizontal transfer of therapeutic antimiR

Parastoo Pourali[1], Eva Neuhöferová[1], Behrooz Yahyaei[2,3], Milan Svoboda[4] , Adéla Buchnarová[5] and Veronika Benson[5*]

[1]Institute of Microbiology, Czech Academy of Sciences, Czech Republic

[2]Department of Medical Sciences, Sha.C., Islamic Azad University, Shahrood, Iran

[3]Department of Medical Sciences, Nanoparticle Research Center in Medicine, Sha.C., Islamic Azad University, Shahrood, Iran

[4]Institute of Analytical Chemistry, Czech Academy of Sciences, Brno, 602 00, Czech Republic

[5]Faculty of Health Studies, Technical University of Liberec, Liberec, Czech Republic

[*]Email: veronika.benson@tul.cz

Protein content in EVs vs. EV-AuNPs

Bradford assay

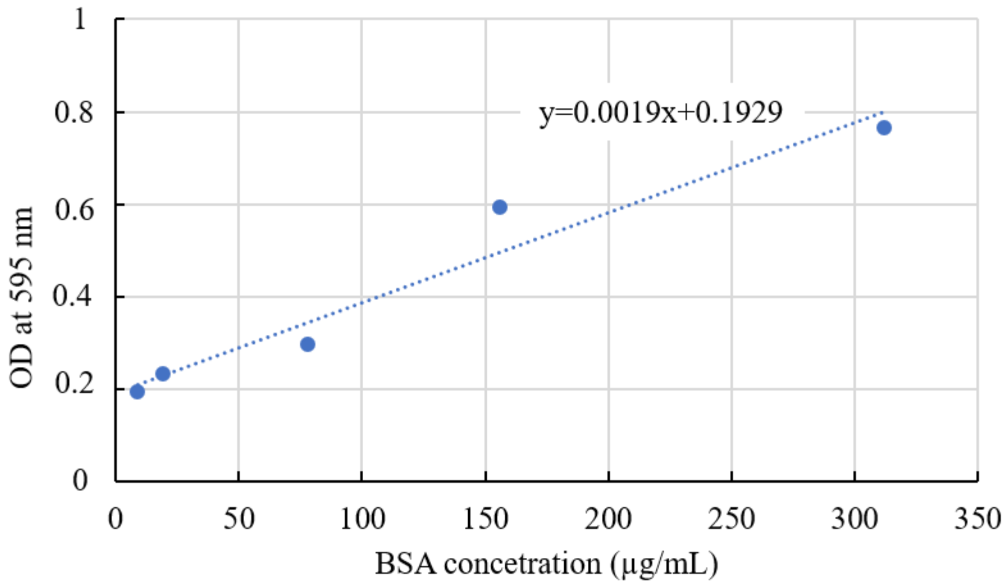

Supplementary Figure 1. BSA standard curve using known amounts of BSA.

qPCR analysis of miR-135b levels

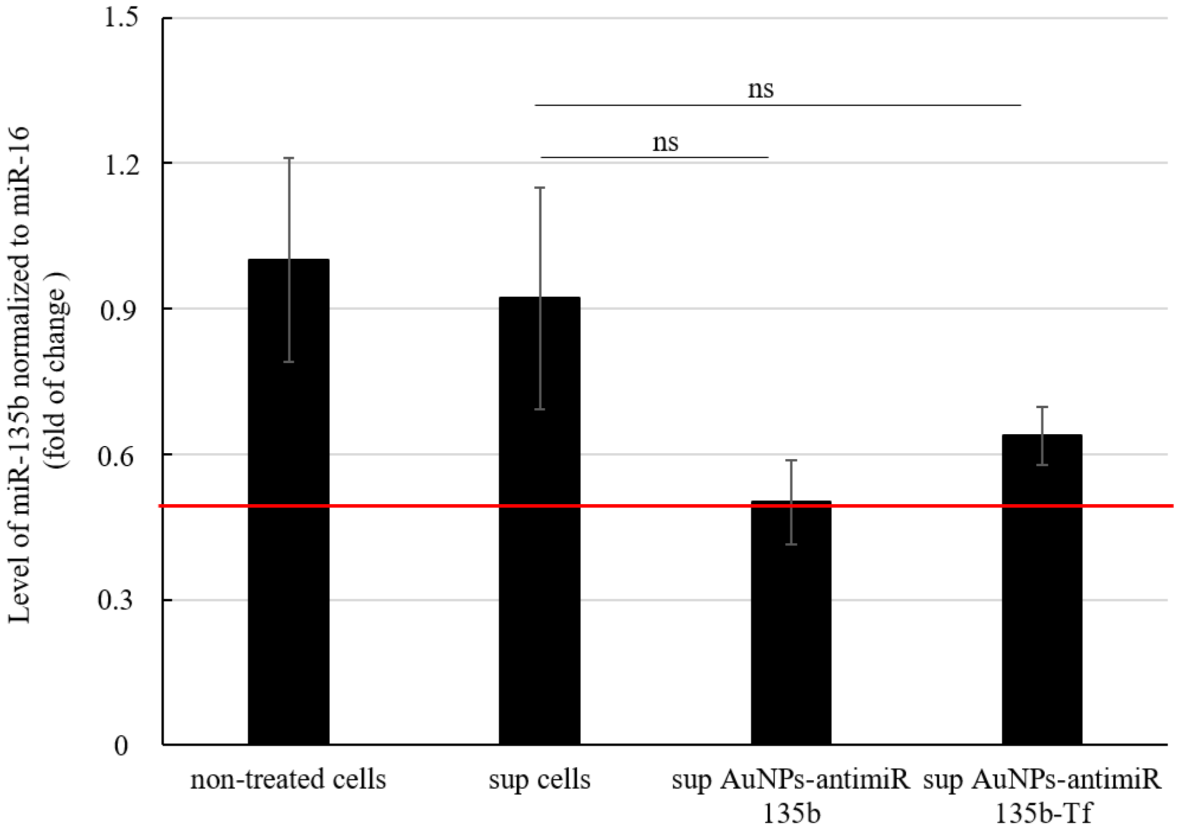

Supplementary Figure 2. The inhibitory effect of antimir 135b delivered into 4T1 tumor cells via EVs-entrapped AuNPs. Differences in miR-135b level among the control non-treated cells and conditioned acellular media (supernatants) collected from cell culture after EVs production and before their extraction. Level of target miR-135b was normalized using miR-16 internal control and the fold of change was assessed by standard 2^{-ddCt} algorithm. The red line marks fold of change = 0.5. ns stands for non-significant.

The miR-135b level was similar in both, the non-treated cells and the cells treated with conditioned media of 4T1 cells incubated with empty EVs (sup cells). Application of conditioned media derived from cells incubated with EVs-AuNPs-antimiR 135 b with or without Tf (sup AuNPs-antimiR 135b-Tf or sup AuNPs-antimiR 135b) led to modest reduction of themiR-135b. This decrease was borderline and exceeded the 0.5 cut off to reach significance given by the standard 2^{-ddCt} calculation.
